# A Modified Technique for Applying Closed Incision Negative Pressure Therapy Dressing Following Total Joint Arthroplasty

**DOI:** 10.7759/cureus.20539

**Published:** 2021-12-20

**Authors:** Shane Dowling, Timothy B Alton

**Affiliations:** 1 Adult Reconstruction, Proliance Orthopedic Associates, Renton, USA; 2 Orthopaedics, Valley Medical Center, Renton, USA

**Keywords:** orthopedics, arthroplasty, hip replacement, knee joint, closed incision negative pressure therapy, cinpt, incisional negative pressure wound therapy

## Abstract

Postoperative incisional management subsequent to total joint replacement arthroplasty is of importance to the orthopedic surgical team. The application of closed incision negative pressure therapy (ciNPT) to surgical incisions following replacement arthroplasty has demonstrated positive outcomes in orthopedics. This paper describes a technique involving the postoperative application of ciNPT over closed incisions originating from joint arthroplasty to facilitate a reduction in the incidence of surgical site complications (SSCs). To address any potential challenges that may be associated with ciNPT application and removal, the ciNPT dressing was applied to the knee incision with approximately 15 degrees of ﬂexion utilizing the total knee bump to allow the knee to rest with ﬂexion at that angle. For posterior hip replacements or revisions, the readily adjustable ciNPT dressing was enlisted for use to cover curvilinear incisions. The adhesive drape over the foam ciNPT dressing would be blocked to ensure that drain placement, if used, would not be incorporated with the hydrocolloid portion of the dressing. In order to properly apply the dressing, it was imperative that the hydrocolloid portion was not subject to any buckling. The dressing was walked over the foam ciNPT dressing to ensure that there was an absence of tension on the dressing. The manufacturer’s instructions support dressing use for a maximum of seven days with continuous subatmospheric pressure (-125 mmHg) applied to the closed incision. Applying the adhesive ciNPT drape over the ciNPT foam dressing with a minimal amount of tension is integral to attaining positive outcomes using ciNPT. Employing ciNPT may reduce the risk of delayed incisional healing and SSCs, which may alleviate providers from extra postoperative global visits.

## Introduction

Total arthroplasty procedures involving either the knee or hip are common orthopedic interventions that generally yield positive outcomes. The rise in these procedures is due in part to the elevated incidence of osteoarthritis and the growing aging population [1]. Based upon 2000 to 2014 data, primary total hip arthroplasty (THA) and total knee arthroplasty (TKA) are projected by 2030 to grow 71% (635,000 procedures) and 85% (1.26 million procedures), respectively [2].

Despite the effectiveness of these orthopedic interventions, diverse postoperative complications are common occurrences following primary knee and hip arthroplasty [3]. Surgical site complications (SSCs) may encompass delayed incision healing, protracted drainage at the surgical defect, the development of seromas and/or hematomas, suture-associated abscesses, and surgical site infections (SSIs) [3-4].

Postoperative swelling or edema is a common SSC associated with increased nociception, reduced range of motion, gait alteration, decreased strength of the quadriceps, and delayed recovery [3-5]. In obese THA or TKA patients, generalized peripheral edema is noted as a common postoperative SSC and is deemed to be of importance to mitigate postoperative edema in order to facilitate the optimization of the quality of care as well as manage resource utilization [5-6]. Postoperative lower extremity edema may result in decreased functional performance and can have an adverse impact on inpatient length of stay (LOS) and influence the patient’s perception of the surgical outcome [1,5].

Protracted wound drainage following total joint arthroplasty presents an elevated risk for developing a periprosthetic joint infection or an SSI [4,7-9]. While the incidence of periprosthetic infections post-arthroplasty is generally small, the risk of superficial or deep infections is significant at 0.2% and 2.2% [10-11]. With the presentation of SSIs, the following are elevated: inpatient LOS, readmission, surgical revision, and reimplantation of prostheses [12]. It should be noted that if a patient is undergoing a surgical revision of a previous arthroplasty procedure, there is a greater risk of SSC, SSI, and inpatient readmission [6]. The role of SSCs in postoperative recovery is frequently underplayed even though SSCs represent a leading cause of unscheduled early readmission [3,13-14].

Closed incision negative pressure therapy (ciNPT) has been reported to decrease the incidence of SSC such as incisional dehiscence, hematoma, and seroma, and sequester the wound environment to reduce the incidence of SSI [3,6,15]. This paper describes a technique involving the postoperative application of ciNPT over closed incisions originating from joint arthroplasty to facilitate a reduction in the incidence of SSCs.

## Materials and methods

**Figure 1:**
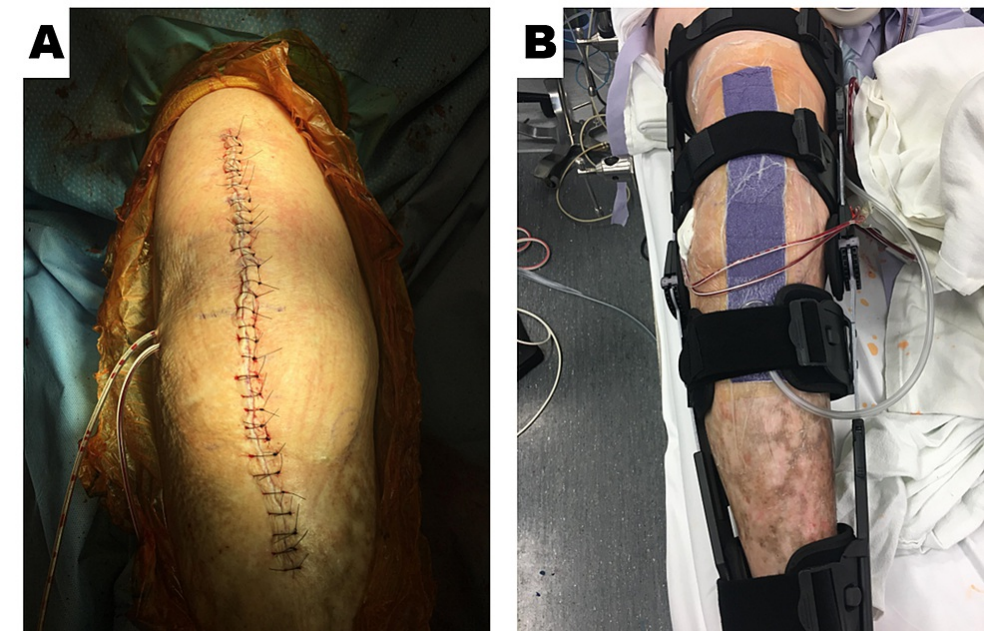
Representative image depicting knee incision post total joint arthroplasty before and after the application of a closed incision management system using negative pressure therapy. A. Closure of incision post knee arthroplasty with sutures. B. Post knee arthroplasty with ciNPT dressing ciNPT = closed incision negative pressure therapy.

This was an observational study documenting the initial experience with commercially available, ciNPT (3M™ Prevena™ Incision Management System; 3M, San Antonio, TX). All patients provided written informed consent for their photographs and data to be used for research and publication purposes. The study was conducted in accordance with the World Medical Association Declaration of Helsinki's ethical guidelines. The surgeon at a single center exercised their professional discretion in selecting the arthroplasty patients that would receive ciNPT following TKA or THA. Prior to the arthroplasty procedure, the preoperative evaluation included radiography to image the region of interest. The author’s institution adhered to a surgical care improvement plan (SCIP), which mandates the administration of perioperative antibiotics for 24 hours. During the arthroplasty procedure, irrigation of the surgical wound was performed via jet lavage using 0.05% chlorhexidine gluconate in sterile water (Irrisept® System; Irrimax Corporation, Gainesville, FL). The topical irrigant was allowed to dwell within the surgical defect for 3 minutes before being aspirated. Following the surgical intervention, closure of the arthroplasty incision was achieved with sutures. Immediately postoperatively within the operating theater and while operative drapes were in place, either a dressing readily adjustable by the surgeon (3M™ Prevena™ Customizable System Kit; 3M, San Antonio, TX) or a dressing ready for application (3M™ Prevena™ Peel and Place System Kit; 3M, San Antonio, TX) were applied sterilely to the surgical incision. The ciNPT dressing was applied to the knee with approximately 15 to 20 degrees of flexion utilizing the total knee bump to allow the knee to rest with flexion at that angle (Figure 1). Specifically, if the readily adjustable ciNPT dressings were used, 1 cm proximal and 1 cm distal to the incision was covered.

**Figure 2:**
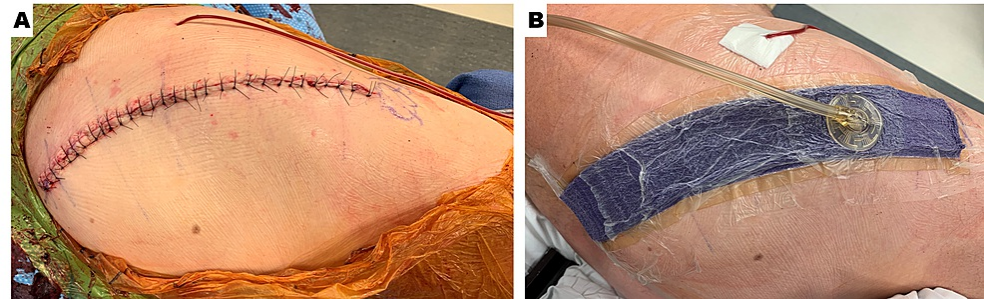
Representative image depicting posterior hip incision post total joint arthroplasty before and after the application of ciNPT A. Closure of incision post hip arthroplasty with sutures. B. Post hip arthroplasty with ciNPT dressing ciNPT = closed incision negative pressure therapy

Additionally, the adhesive over the foam ciNPT dressing would be blocked to ensure that the drain placement (if applicable) was not incorporated with the hydrocolloid portion of the dressing. For posterior hip replacements or revisions, the readily adjustable ciNPT dressing was enlisted for use to cover curvilinear incisions. In order to properly apply the dressing, it was imperative that the hydrocolloid portion was not subject to any buckling. Further, the drape was walked over the foam ciNPT dressing to ensure that there was an absence of tension on the dressing. Similar to the protocol for coverage of the TKA incision, 1 cm proximal and 1 cm distal to the THA incision was covered by the readily adjustable ciNPT foam dressing (Figure 2).

The manufacturer’s instructions for use support dressing use for a maximum of seven days. Incisional management was achieved via seven days of ciNPT, which applied subatmospheric pressure (-125 mmHg) to the clean closed incision. Upon completion of the arthroplasty procedure, patients were transferred to the surgical intensive care unit for recovery. ciNPT was initially applied with a large commercially available unit restricted for inpatient use, but after 24 hours, the patients transitioned to the portable, AC-powered unit without changing the dressing. Postoperative visits during the global period were scheduled for either two, three, or six weeks. Suture removal occurred 14 to 17 days post-procedure.

Case 1

Patient 1 was a 62-year-old male, who received a right hip revision to address osteolysis and polyethylene wear. The patient presented with no known comorbidities and previously underwent a revision. He was prescribed cephalexin prophylactically and received a posterior incision measuring 25 cm in length. Upon the completion of the hip revision, a foam dressing (Prevena Customizable System Kit) was applied immediately intraoperatively within the operating theater. ciNPT was applied to the foam dressing and remained in place for seven days. The length of stay (LOS) was one day. After seven days, the incision was evaluated and remained closed at the 30-day follow-up.

Case 2

Patient 2 was a 65-year-old male, who received a right hip arthroplasty (RHA). The patient presented with the following comorbidities: hypertension and obesity (BMI = 42 kg/m2) He was prescribed cephalexin prophylactically and received an anterior incision measuring 14 cm in length. Closure of the incision entailed the use of running subcuticular sutures. Upon the completion of the hip arthroplasty, a foam dressing (Prevena Peel and Place System Kit) was applied immediately intraoperatively within the operating theater and remained in place for seven days. The LOS was one day. After seven days, the incision was evaluated and remained closed at the 30-day follow-up.

Case 3

Patient 3 was an 85-year-old male who received a total knee revision as an intervention for extensor mechanism disruption. The patient presented with the following comorbidity: coronary artery disease. The patient was noted to have had three previous total knee revisions. He received intravenous vancomycin for six weeks and oral levofloxacin for possible infection versus culture contamination. The patient received a midline incision measuring 30 cm in length, and closure of the incision entailed the use of sutures. Upon the completion of the TKA, a foam dressing (Prevena Customizable System Kit) was applied immediately intraoperatively for seven days. This had a LOS of seven days. After seven days, the ciNPT dressing was removed and the incision was evaluated. The incision remained closed at the 30-day follow-up.

Case 4

Patient 4 was a 63-year-old male who received a total knee replacement for osteoarthritis. The patient presented with the following comorbidities: chronic obstructive pulmonary disease (COPD), obesity (BMI = 37 kg/m^2^), and tobacco use. The patient received a midline incision measuring 20 cm in length, and closure of the incision entailed the use of sutures. Upon the completion of the TKA, a foam dressing (Prevena Customizable System Kit) was applied immediately intraoperatively for seven days. This had a LOS of two days. After seven days, the ciNPT dressing was removed and the incision was evaluated. The incision remained closed at the 30-day follow-up.

Case 5

Patient 5 was an 86-year-old female who received a revision total knee replacement for aseptic loosening of her components. The patient presented with the following comorbidity: hypertension, diabetes, rheumatoid arthritis, obesity (BMI = 43 kg/m^2^), depression, kidney disease, and asthma. The patient received a midline incision measuring 20 cm in length, and closure of the incision entailed the use of sutures. Upon the completion of the TKA, a foam dressing (Prevena Customizable System Kit) was applied immediately intraoperatively for seven days. She was prescribed cephalexin prophylactically. This had a LOS of two days. After seven days, the ciNPT dressing was removed and the incision was evaluated. The incision remained closed at the 30-day follow-up.

## Results

This pilot study included one female (20.0%) patient and four male (80.0%) patients, who were at an elevated risk for developing SSC and received ciNPT intraoperatively following total joint arthroplasty (Table 1).

**Table 1:** Patient characteristics, arthroplasty type, and postoperative outcomes ciNPT = closed incision negative pressure therapy; LOS = length of stay; SSC = surgical site complications; HTN = hypertension; BMI = body mass index; COPD = chronic obstructive pulmonary disease; DM = diabetes mellitus; RA = rheumatoid arthritis; CAD = coronary artery disease

Patient	Age	Sex	Comorbidities	Arthroplasty Location	Arthroplasty Revision	ciNPT Duration	Dressing Changes	LOS	SSCs w/in 30 days	Swelling	Closed at 30 days?	Total Postoperative Visits During Global Period
1	62	M	n/a	Hip	Yes	7 days	0	1 day	0	No	Yes	3
2	65	M	HTN and obesity (BMI = 42 kg/m^2^)	Hip	No	7 days	0	1 day	0	No	Yes	2
3	85	M	CAD	Knee	Yes	7 days	0	7 days	0	No	Yes	2
4	63	M	COPD, obesity (BMI = 37 kg/m^2^), tobacco use	Knee	No	7 days	0	2 days	0	No	Yes	2
5	86	F	HTN, DM, RA, obesity (BMI = 43 kg/m^2^), depression, kidney disease, asthma	Knee	Yes	7 days	0	2 days	0	No	Yes	2

The mean age of the patients within our study population was 72.2 ± 12.2 years. Body mass index (BMI) for the population ranged from 21-43 kg/m^2^ (mean = 33.6 ± 10.0 kg/m2). Two procedures were total hip arthroplasty (THA; 4.0%), and three procedures were total knee arthroplasty (TKA; 60.0%). Of the population, three patients (60.0%) had undergone a prior revision. Common comorbidities included obesity (BMI > 30 kg/m^2^; 60%) and hypertension (40%). In our study, none of the patients included had a history of diabetes or glycemic control. Either a customizable (n = 4) or a peel and place (n = 1) polyurethane foam dressing impregnated with 0.019% ionic silver in the interface layer were applied to the sutured incisions (length: 10 - 30 cm). ciNPT was applied across the closed incision for a maximum of seven days. Surgical drains were used to help decrease a joint hematoma and/or seroma. These were removed prior to the patients' discharge from the hospital. Among our study population, no patients (0.0%) experienced SSC within 30 days following the primary arthroplasty or revision procedure. The patients did not require any further application of ciNPT beyond the single, seven-day application of the ciNPT dressing. No continuous passive motion machine was utilized during the postoperative rehabilitation protocol. Four patients were all mobile weight-bearing as tolerated immediately after surgery and range of motion (ROM) as tolerated. However, the lone exception was the patient who underwent an extensor mechanism disruption after their TKA who was non-weight-bearing and was not allowed any ROM for 12 weeks. Additionally, no further antibiotics were prescribed as no signs of infection were noted postoperatively in these patients. In all patients (100.0%), the incision had attained closure by postoperative day 30. The mean length of stay (LOS) for the study population was 2.6 ± 2.5 days. The mean of postoperative visits during the global period was 2.2 ± 0.45 visits.

## Discussion

While ciNPT can be an effective modality, applying the system is not without challenges. To proactively address any potential challenges that may be associated with ciNPT application and removal in high-risk patients, we devised the reported technique for ciNPT placement to minimize tension across the skin edges of the sutured incision.

The genesis of applying the ciNPT dressing on a TKA and TKR with the addition of a total knee “bump” as described within the methods came via trial and error. When using ciNPT over a mobile joint, such as the knee, we found that 15- to 20-degree flexion when applying the drape allowed for minimal restriction on ROM while also minimizing any bucking of the dressing that could cause a leak in the system. This technique also minimized patients’ pain with ROM and any blistering from the tension of the patients’ skin from the drape.

Prior to developing this technique, it was necessary to have personnel within the operating theater to assist by holding the knee in a flexed position during placement of the ciNPT dressing. This entailed needless resource utilization, but then a total knee “bump” was incorporated into the process. Incorporating a total knee “bump” facilitated a similar degree of flexion to the knee and afforded the patient sufficient range of motion without excessive tension across the ciNPT drape perimeter.

This technique ensured that there was no space between the foam dressing and the drape. From the author’s observations, the adoption of this technique has resulted in improved surgical healing time; a decrease in tissue sensitivity along the perimeter of the dressing, specifically the drape; reduced reliance on alternative dry dressings and concomitant changes; and improved postoperative pain upon dressing removal. Applying the adhesive ciNPT drape over the ciNPT foam dressing with a minimal amount of tension is integral to attaining positive outcomes using ciNPT.

The literature has reported positive orthopedic outcomes with ciNPT in TKA/THA. In a randomized control trial (RCT) by Manoharan et al., 57 primary knee arthroplasty procedures in 33 patients were included to assess the effect of ciNPT on quality of life, SSCs, and health economics following TKA. In their study, 12 patients (3 bilateral and 9 unilateral TKAs) received conventional dry dressings and 21 (all bilateral TKAs) had either side randomized to receive either conventional dry dressings (CDD) or ciNPT. Two patients experienced complications; one patient, who received ciNPT demonstrated a medical adhesive-related skin injury as a result of drape application and one patient in the CDD cohort demonstrated persistent drainage that was addressed by applying NPWT.

In a study by Anatone et al., consisting of 323 consecutive primary joint arthroplasty patients, patients were risk-stratified into a couple of sub-groups contingent upon their perceived risk of developing an SSC, postoperatively. Thirty-eight percent (n = 123 patients) were designated as high risk and received a ciNPT dressing following total joint arthroplasty. Sixty-two percent (n = 200) were designated as low risk and received a standard postoperative dressing that does not rely on the administration of subatmospheric pressure. Both sub-groups were subject to incisional closure via monofilament subcuticular suturing, and the postoperative dressings were left in place for a minimum of seven days. The primary outcome evaluated was any postoperative SSC (dehiscence, suture granuloma, protracted drainage, hematoma, and CDC-defined SSI) that necessitated an intervention within 90 days, postoperatively. They noted in comparison to historical controls, a modest yet statistically significant improvement in superficial SSCs following the implementation of risk-stratification (12.0% vs. 6.8%; p = .013). When high-risk patients were treated with ciNPT dressings, there was an appreciable improvement in SSCs relative to historical high-risk controls (26.2% vs. 7.3%; p < .001). Patients designated as low risk in the study group, who were treated with standard postoperative dressings, did not demonstrate any significant improvement relative to historical low-risk controls (8.6% vs. 6.5%; p = .344) [3].

Recently, Cooper and associates utilized a retrospective cohort design to evaluate the effect of ciNPT (n = 27) on the rate of deep SSI, incisional complications, and reoperation post periprosthetic fractures following total joint arthroplasty versus an antimicrobial Hydrofiber dressing (n = 40). Patients treated with ciNPT demonstrated fewer SSCs (4% vs. 35%; p = .002) and fewer deep infections (0% vs. 25%; p = .004) and reduced the incidence of reoperations pertaining to the surgical site (4% vs. 25%; p = .021) compared to patients receiving antimicrobial dressing (AMD). It should be noted that the manufacturer’s instructions for use support a maximum of seven-day usage for ciNPT dressing; however, in the Cooper study, the mean therapy duration was 9.3 days for AMD versus 8.5 days for ciNPT (p = .825). Nevertheless, the rates of SSCs exhibited statistical differences between the cohorts in this study assessing the application of ciNPT in the postoperative management of patients following surgical procedures for periprosthetic fractures [16].

In a prospective RCT at a single center, 159 total joint arthroplasty patients undergoing a surgical revision were included for analysis. The study by Newman and colleagues assessed the use of ciNPT against a control group that received a silver-impregnated occlusive dressing in at-risk THA or TKA patients. Wound complications as a primary outcome and readmission and reoperation rates as secondary outcomes were collated within 12 weeks of the revision postoperatively. The rate of postoperative complications associated with the surgical defect was significantly lower in the group that was administered ciNPT (8 [10.1%] vs. 19 [23.8%]; p = .022). No significant difference was noted in readmission rates between ciNPT and the silver-impregnated occlusive dressing cohorts (16 [20.3%] vs. 19 [23.8%]; p = .595). The rate of reoperation was lower in the ciNPT cohort relative to the control cohort (2 [2.5%] vs. 10 [12.5%]; p =.017). When adjusting for inflammatory arthritis and a prior history of periprosthetic joint infection, the ciNPT cohort exhibited a significantly decreased rate of SSCs (OR 0.28; 95% CI .11 - .68) [12].

In addition to these studies, other RCTs and meta-analyses have reported a significant reduction in the incidence of SSIs using ciNPT.17-19 In an analysis of 188 patients (ciNPT intervention = 98 versus Control = 90), Gombert et al noted patients with peripheral artery disease who underwent vascular surgery and received ciNPT had a significantly reduced rate of SSI relative to control patients (13.2% versus 33.3%; p = .0015) [17]. In two different meta-analyses by Singh and colleagues, outcomes were evaluated between ciNPT and conventional dressing and between two closed incision management (CIM) systems [18-19]. Treatment effects were concerned with the presence (or absence) of SSI. The first study initially identified 540 publications and included 30 investigations for analysis. In sensitivity analyses using random-effects models, all analyses were significant (p < .05) save obstetrics (p = .11) [18]. The second study extracted 408 publications, but 17 studies were selected for the final analyses [19]. In this study, two meta-analyses were performed to evaluate different CIM systems. Meta-analysis 1 evaluated a ciNPT system using a foam dressing versus conventional dressing (n = 9 studies), and Meta-analysis 2 evaluated a ciNPT system using a multilayer absorbent dressing versus conventional dressing (n = 8 studies). Meta-analysis 1 demonstrated that the control cohort was 3.17 times more likely to develop SSI relative to the cohort that received the ciNPT system using a foam dressing (weighted mean OR 3.17; 95% CI 2.1 to 4.65; p < .0001). Meta-analysis 2 results were nonsignificant relative to SSI rates between the cohort that received the ciNPT system using a multilayer absorbent dressing and the control (weighted mean OR 1.70; 95% CI 0.94 to 3.08; p = .08) [19]. Despite the small sample size, none of our patients developed an SSI following the primary arthroplasty or revision procedure. Patients were further seen at an eight-week, 12-week, and one-year follow-up to monitor for infection.

One limitation of this report is the small case series size. Another potential limitation is selection bias. However, this might be mitigated as the use of ciNPT to manage surgical incisions following total joint arthroplasty is our institution’s policy. While a reduction of swelling or edema was noted, we did not measure postoperative swelling by either bioimpedance spectroscopy [5] or lymphography using Indocyanine green to measure lymphedema. Future studies would entail increasing the sample size alongside appropriate controls. Such future studies would also encompass robust statistical analysis to assess the clinical efficacy of ciNPT relative to the standard of care control as well as to gauge cost-effectiveness for health economic considerations.

## Conclusions

In our study, we focused on the technique of applying ciNPT following primary joint arthroplasty or arthroplasty revisions. This work demonstrated that incisional management with ciNPT for seven days may facilitate a reduced incidence of surgical site complications. In our experience, SSCs were reduced by utilizing ciNPT and were integral in edema management postoperatively in these five patients. Our patients treated with ciNPT had a mean length of stay of 2.6 ± 2.5 days. All incisions remained closed by postoperative day 30, and we noted no complications within 30 days following the primary arthroplasty or revision procedure.
